# What is next for occupational cancer epidemiology?

**DOI:** 10.5271/sjweh.4067

**Published:** 2022-10-29

**Authors:** Michelle C Turner

**Affiliations:** 1 Barcelona Institute for Global Health (ISGlobal), Barcelona, Spain; 2 Universitat Pompeu Fabra (UPF), Barcelona, Spain; 3 CIBER Epidemiología y Salud Pública (CIBERESP), Madrid, Spain

Research on occupational causes of cancer has identified 47 known (Group 1) agents associated with 23 types of cancer through 2017, an increase from 28 agents in 2004 (1, 2). Occupational agents include chemicals and chemical mixtures; radiation and radionuclides; airborne particles and complex mixtures; and metals and metal compounds. The global burden of cancer due to 14 of the Group 1 agents was estimated to total 349 000 [95% uncertainty interval (UI) 269 000–427 000] deaths in 2016, or 3.9% (95% UI 3.2–4.6%) of all cancer deaths, including 299 998 or 17.6% (95% UI 13.8–21.3%) of lung cancer deaths (3). There are also exposures in various occupations, industries, or processes classified as Group 1 where the causal agent is not necessarily identified. In 2022, occupational exposure as a firefighter was most recently classified in Group 1, with sufficient evidence among humans for mesothelioma and bladder cancer, and limited evidence for other cancers (4).

Despite achievements in identifying occupational causes of cancer, a range of research needs remain, including identifying additional cancer sites for Group 1 agents and more definitive studies for exposures where the evidence among humans remains limited or inadequate (1, 5–7). There may be outstanding methodological concerns or findings that are inconsistent or of poor quality or informativeness.

Research recommendations to address classification uncertainties for 20 priority occupational agents have been detailed (8). They include conducting new epidemiological studies in highly exposed occupations or populations, improving (quantitative) exposure assessment including through biomarkers of exposure, enhancing statistical power through extended follow-up or pooled studies, and furthering human mechanistic studies. High quality human mechanistic evidence can provide valuable information when epidemiology studies are not available or feasible (5). A 2019 Advisory Group considered 170 different agents in terms of their suitability for (re-)evaluation with a range of chemical, metal, or complex exposures of relevance for occupational settings prioritized based on new human epidemiology, mechanistic and/or cancer bioassay data (9). 

There have been calls to update existing cohorts when valuable follow-up time has accrued allowing investigation of the full potential impact of exposures on health (10). There is a longstanding need for occupational epidemiological and exposure assessment studies in low- and middle-income countries, where there are often few or no available studies and exposure levels maybe higher (11–14). There may also be differences in working conditions, exposure patterns, and worker protections (15, 16). Research challenges include declines in participation rates, funding, and research infrastructures (1, 17–19).

In parallel, epidemiological research has innovated over time to comprise increasingly larger-scale prospective cohort studies and consortia, use of electronic data linkage, causal inference methods and triangulation of evidence, reinforcing the ongoing utility of observational research methods (20). The recent COVID-19 pandemic has reinforced the need for a global perspective in epidemiological research, multidisciplinarity, and broadening perspectives regarding fundamental underlying determinants of health (21, 22). There have also been calls for greater equity and inclusiveness in health research, both in Europe, and worldwide (23, 24).

Efforts to stimulate future research and investment in occupation and cancer may benefit from the use of new rapidly evolving research methods, closer alignment with global public health priorities, and strengthening of international partnerships supporting excellence and inclusiveness in research. For example, a range of methodological advancements have emerged from application of exposome concepts in epidemiology. In Europe, birth cohort consortia seeking to characterize the early-life exposome, as well as other efforts, have driven much innovation (25, 26). The exposome concept was proposed in 2005 to stimulate investment to better characterize environmental exposures throughout the lifecourse using novel technologies, offering a complementary perspective to that of the genome (27). Although occupational exposure has previously not been emphasized, research in the internal and external occupational exposome is now beginning to emerge (28).

A range of statistical methods for analysis of multiple correlated exposures have advanced (29). Extended Bayesian profile regression mixture (PRM) models have been used to examine multiple highly correlated ionizing radiation exposures for lung cancer risk among miners (30). An exposome-wide association study examined a range of personal and occupational factors in B-cell lymphoma, suggesting that single-factor research approaches maybe suboptimal for new disease insights (31). There are exposome technologies for personal monitoring of workers (28, 32) and other novel research methods including natural language processing and text mining (33, 34), automated network assembly approaches to summarizing literature (35), efforts to combine epidemiological data with those from other evidence streams (36), and new technologies to facilitate secure decentralized pooled analyses of data (37).

However, there is an ongoing need for continued efforts to better characterize the occupational and corresponding non-occupational exposome over the lifecourse. Research priorities include establishing new cohort studies with appropriate biosample collection, improved questionnaire and personal monitoring data, increasing multidisciplinary collaboration to utilize innovative data and methods, and integrating genetic data in exposome studies for causal inference (38-40). At the same time, careful consideration of the policy relevance of exposome studies remains of importance (41), as are continued efforts in conventional epidemiological case–control and cohort studies in occupation and cancer (8). Occupational studies examining exposures of relevance for the general population may favor greater investment (42). There are environmental exposure routes for occupational agents, bystander or spousal exposure to occupational agents, and potential transgenerational health effects (43–45). Birth cohorts may represent a typically underused resource for research in occupation and health (46). Principles for safeguarding integrity in environmental and occupational research have also been outlined (47).

Research on occupation and cancer may benefit from closer alignment with recent high-profile initiatives on related topics as well as with global public health priorities. The United Nations Sustainable Development Goals note the need for decent work (48). The EU Strategic Framework on Health and Safety at Work describes the elimination of work-related deaths by 2030 and reduced illness through improved data collection, updated rules on hazardous substances, including those of relevance for renewable energy technologies (ie, lead, cobalt) or of asbestos exposure in building renovations for greening, increased health literacy at work, and adapting working conditions for patients (49). A large-scale survey of worker exposure to cancer risk factors is being implemented in Europe to collect standardized data across different European countries (50). The Health Environment Agenda for Europe project defined priority areas for research on rapidly changing work and employment conditions, climate change and worker health, working time and long working hours, ageing workers, and neglected work-related diseases (51).

Rapidly changing work conditions were exemplified during the COVID-19 pandemic, with potential direct or indirect effects on health and cancer. Shifts in overall global cancer research focus were also described (52). Increasing unemployment and economic downturns in high- and middle-income countries have been associated with increased cancer mortality for treatable cancers, with less access to healthcare underlying findings (53). There is also increasing interest in precarious employment and potential direct or indirect impacts on health and quality of life (54).

Public health efforts directed at catching-up in cancer screening and on improving health systems and public health literacy following the COVID-19 pandemic may offer opportunities to advance cancer control and improved health literacy at work (55, 56). Further, there may greater opportunities for strengthened clinical partnerships for occupational epidemiologists. For example, dramatic gains in survival due to early detection have been demonstrated for lung cancer (57). However, occupational (or environmental) exposures are not systematically incorporated into screening algorithms, and further research and collaboration with clinical partners is warranted (58–61).

The potential importance of climate change in cancer, including occupation and cancer, may also not be fully understood (62, 63). Climate change may relate to increasing exposure to environmental or occupational carcinogens, including air pollution, adverse dietary exposures, changes in physical activity levels, ultraviolet radiation, water pollution, infections, and parasites due to extreme weather events, wildfires (4), heat, sea-level rise, and changes in land-use. There may also be disruptions in cancer care. Climate change may further exacerbate existing socioeconomic inequities, and social determinants of cancer. Increasing occupational heat stress is related to acute and chronic health effects as well as reduced productivity (64–66). Studies of occupational heat exposure and cancer risk are beginning to emerge (67). Interventions to jointly address climate change and disease prevention, including cancer, have been proposed (68). There are also new agents rapidly entering the workplace where little is known regarding their carcinogenicity to humans. A planetary health perspective suggests that humanity is outside the safe operating space of the planetary boundary, with increasing production and release of chemical industry production exceeding the ability to conduct safety assessments (69).

Lastly, strengthened international partnerships are critical for future advances in occupational cancer research. Efforts in coordination of European birth cohort studies, and later occupational cohort studies, have led to major advancements in research and inclusiveness (25, 26, 70, 71). Network funding initiatives provide valuable support and developed out of a recognized need to increase equitable access to funding and research infrastructures (72). A recent example is the OMEGA-NET COST Action, which sought to improve coordination and harmonization of European occupational cohort studies by connecting researchers through a range of research coordination and capacity building activities, with a particular focus on connecting researchers in traditionally less research-intensive countries (71). Online inventories of occupational cohort studies and exposure assessment tools were developed (73, 74) as were advancements in theoretical frameworks, consensus definitions and recommendations for future research on emerging topics in occupational health (54, 75, 76).

The need for occupational epidemiological and exposure monitoring studies in low- and middle-income countries has long been recognized. Priorities for cancer research in low- and middle-income countries have recently been described as separate to those of high-income countries, and highlighted the need to reduce the burden of patients presenting with advanced-stage disease, primary prevention and early detection, and innovative and affordable technology in cancer control (77). Documenting and minimizing exposure to established occupational carcinogens is critical to prioritize interventions and prevent future cancer burden (11, 34, 78). Generating country-specific evidence for effective prevention may be helpful in this regard (77). Research questions on cancers that are of local importance, using appropriate research methods for available infrastructure, and partnerships for mutually rewarding collaborations have been described (77, 79). However, cancer registry and infrastructure challenges have been outlined, including of poor-quality data and an absence of legal frameworks for cancer registration (19). International collaboration has had demonstrated impacts in research and capacity building, though sustained political and financial commitment is needed (16, 80, 81).

The occupational epidemiology community has a great opportunity to promote new efforts in occupation and cancer while at the same time reducing inequalities in health and research.

## References

[1] Loomis D, Guha N, Hall AL, Straif K (2018). Identifying occupational carcinogens:an update from the IARC Monographs. Occup Environ Med.

[2] Siemiatycki J, Richardson L, Straif K, Latreille B, Lakhani R, Campbell S (2004). Listing occupational carcinogens. Environ Health Perspect.

[3] GBD 2016 Occupational Carcinogens Collaborators Global and regional burden of cancer in 2016 arising from occupational exposure to selected carcinogens:a systematic analysis for the Global Burden of Disease Study 2016 (2020). Occup Environ Med.

[4] Demers PA, DeMarini DM, Fent KW, Glass DC, Hansen J, Adetona O (2022). Carcinogenicity of occupational exposure as a firefighter. Lancet Oncol.

[5] IARC Preamble to the IARC Monographs, amended January 2019.

[6] Marant Micallef C, Shield KD, Baldi I, Charbotel B, Fervers B, Gilg Soit Ilg A (2018). Occupational exposures and cancer:a review of agents and relative risk estimates. Occup Environ Med.

[7] IARC Agents Classified by the IARC Monographs, Volumes 1-132.

[8] Ward EM, Schulte PA, Straif K, Hopf NB, Caldwell JC, Carreón T (2010). Research recommendations for selected IARC-classified agents. Environ Health Perspect.

[9] IARC (2019). Monographs Priorities Group Advisory Group recommendations on priorities for the IARC Monographs Lancet Oncol.

[10] Vainio H, Weiderpass E (2017). Importance of updating old cohorts for new findings. Occup Environ Med.

[11] Loomis D (2020). Estimating the global burden of disease from occupational exposures. Occup Environ Med.

[12] IARC (2015). Working Group on the Evaluation of Carcinogenic Risks to Humans Outdoor air pollution IARC monographs on the evaluation of carcinogenic risks to humans 109.

[13] IARC (2017). Working Group on the Evaluation of Carcinogenic Risks to Humans Some organophosphate insecticides and herbicides IARC monographs on the evaluation of carcinogenic risks to humans.

[14] IARC (2019). Working Group on the Evaluation of Carcinogenic Risks to Humans Styrene, Styrene-7,8-oxide, and Quinoline IARC monographs on the evaluation of carcinogenic risks to humans.

[15] Groß JV, Fritschi L, Erren TC (2019). Shift work and cancer:more research needed from low and middle income countries. Occup Environ Med.

[16] Hosseini B, Hall AL, Zendehdel K, Kromhout H, Onyije FM, Moradzadeh R (2121). Occupational exposure to carcinogens and occupational epidemiological cancer studies in Iran:a review. Cancers.

[17] Stayner TL, Collins JJ, Guo YL, Heederik D, Kogevinas M, Steenland K (2017). Challenges and opportunities for occupational epidemiology in the twenty-first century. Curr Environ Health Rep.

[18] Sweity S, Sutton C, Downe S, Balaam MC, McElvenny DM (2020). Challenges to and facilitators of occupational epidemiology research in the UK. Health Policy.

[19] Ngwa W, Addai BW, Adewole I, Ainsworth V, Alaro J, Alatise OI (2022). Cancer in sub-Saharan Africa:a Lancet Oncology Commission. Lancet Oncol.

[20] McCullough LE, Maliniak ML, Amin AB, Baker JM, Baliashvili D, Barberio J (2022). Epidemiology beyond its limits. Sci Adv.

[21] No authors listed (2021). How epidemiology has shaped the COVID pandemic Nature.

[22] Hinchliffe S, Manderson L, Moore M (2021). Planetary healthy publics after COVID-19. Lancet Planet Health.

[23] Berner-Rodoreda A, Rehfuess EA, Klipstein-Grobusch K, Cobelens F, Raviglione M, Flahaut A (2019). Where is the 'global'in the Europèan Union's health research and innovation agenta?BMJ Glob Health.

[24] Gallo F, Seniori Costantini A, Puglisi MT, Barton N (2021). Biomedical and health research:an analysis of country participation and research fields in the EU's Horizon 2020. Eur J Epidemiol.

[25] Vrijheid M, Slama R, Robinson O, Chatzi L, Coen M, van den Hazel P (2014). The human early-life exposome (HELIX):project rationale and design. Environ Health Perspect.

[26] Vrijheid M, Basagaña X, Gonzalez JR, Jaddoe VWV, Jensen G, Keun HC (2021). Advancing tools for human early lifecourse exposome research and translation (ATHLETE):Project overview. Environ Epidemiol.

[27] Wild CP (2005). Complementing the genome with an “exposome”:the outstanding challenge of environmental exposure measurement in molecular epidemiology. Cancer Epidemiol Biomarkers Prev.

[28] Pronk A, Loh M, Kuijpers E, Albin M, Selander J, Godderis L (2022). Applying the exposome concept to working-life health:The EU EPHOR project. Environ Epidemiol.

[29] Maitre L, Guimbaud JB, Warembourg C, Güil-Oumrait N, Petrone PM, Chadeau-Hyam M (2022). State-of-the-art methods for exposure-health studies:Results from the exposome data challenge event. Environ Int.

[30] Belloni M, Laurent O, Guihenneuc C, Ancelet S (2020). Bayesian profile regression to deal with multiple highly correlated exposures and a censored survival outcome. First application in ionizing radiation epidemiology. Front Public Health.

[31] Hosnijeh FS, Casabonne D, Nieters A, Solans M, Naudin S, Ferrari P (2021). Association between anthropometry and lifestyle factors and risk of B-cell lymphoma:an exposome wide analysis. Int J Cancer.

[32] Kuijpers E, van Wel L, Loh M, Galea KS, Makris KC, Stierum R (2021). A Scoping Review of Technologies and Their Applicability for Exposome-Based Risk Assessment in the Oil and Gas Industry. Ann Work Expo Health.

[33] Schoene AM, Basinas I, van Tongeren M, Ananiadou S (2022). A Narrative Literature Review of Natural Language Processing Applied to the Occupational Exposome. Int J Environ Res Public Health.

[34] Kromhout H, van Tongeren M, Peters CE, Hall AL (2020). Commentary. Occup Environ Med.

[35] Scholten B, Guerrero Simon L, Krishnan S, Vermeulen R, Pronk A, Gyori BM (2022). Automated network assembly of mechanistic literature for informed evidence identification to support cancer risk assessment. Environ Health Perspect.

[36] Scholten B, Portengen L, Pronk A, Stierum R, Downward GS, Vlaanderen J (2022). Estimation of the Exposure-Response Relation between Benzene and Acute Myeloid Leukemia by Combining Epidemiologic, Human Biomarker, and Animal Data. Cancer Epidemiol Biomarkers Prev.

[37] Marcon Y, Bishop T, Avraam D, Escriba-Montagut X, Ryser-Welch P, Wheater S (2021). Orchestrating privacy-protected big data analyses of data from different resources with R and DataSHIELD. PLoS Comput Biol.

[38] Avery CL, Green Howard A, Ballou AF, Buchanan VL, Collins JM, Downie CG (2022). Strengthening Causal Inference in Exposomics Research:Application of Genetic Data and Methods. Environ Health Perspect.

[39] Jia P, Lakerveld J, Wu J, Stein A, Root ED, Sabel CE (2019). Top 10 Research Priorities in Spatial Lifecourse Epidemiology. Environ Health Perspect.

[40] Vineis P, Robinson O, Chadeau-Hyam M, Dehghan A, Mudway I, Dagnino S (2020). What is new in the exposome?Environ Int.

[41] Health (2022). Council of the Netherlands Relevance of exposome research for policy No.2022/02e, The Hague.

[42] Blair A, Hines CJ, Thoms KW, Alavanja MCR, Beane Freeman LE, Hoppin JA (2015). Investing in prospective cohorts for etiologic study of occupational exposures. Am J Ind Med.

[43] Li S, Cirillo P, Hu X, Tran V, Krigbaum N, Yu S (2020). Understanding mixed environmental exposures using metabolomics via a hierarchical community network model in a cohort of California women in 1960's. Reprod Toxicol.

[44] Louis LM, Lerro CC, Friesen MC, Andreotti G, Koutros S, Sandler DP (2017). A prospective study of cancer risk among Agricultural Health Study farm spouses associated with personal use of organochlorine insecticides. Environ Health.

[45] Vermeulen R, Silverman DT, Garshick E, Vlaanderen J, Portengen L, Steenland K (2014). Exposure-response estimates for diesel engine exhaust and lung cancer mortality based on data from three occupational cohorts. Environ Health Perspect.

[46] Ubalde-Lopez M, Garani-Papadatos T, Scelo G, Casas M, Lissåker C, Peters S (2021). Working life, health and well-being of parents:a joint effort to uncover hidden treasures in European birth cohorts. Scand J Work Environ Health.

[47] Baur X, Soskolne CL, Bero LA (2019). How can the integrity of occupational and environmental health research be maintained in the presence of conflicting interests?Environ Health.

[48] United Nations Transforming our world:The 2030 agenda for sustainable development A/RES/70/1.

[49] EC (2021). EU strategic framework on health and safety at work 2021-2027 Occupational safety and health in a changing world of work. COM/2021/323 final.

[50] EU-OSHA (2022). Worker survey on exposure to cancer risk factors.

[51] HERA Consortium 2021 EU Research Agenda for the Environment, Climate &Health y2021-2030.Final Draft.

[52] Van Hemelrijck M, Lewison G, Fox L, Dnk Vanderpuye V, Murillo R, Booth CM (2021). Global cancer research in the era of COVID-19:a bibliometric analysis. Ecancermedicalscience.

[53] Maruthappu M, Watkins J, Noor AM, Williams C, Ali R, Sullivan R (2016). Economic downturns, universal health coverage, and cancer mortality in high-income and middle-income countries,1990-2010:a longitudinal analysis. Lancet.

[54] Bodin T, Çağlayan C, Garde AH, Gnesi M, Jonsson J, Kiran S (2020). Precarious employment in occupational health - an OMEGA-NET working group position paper. Scand J Work Environ Health.

[55] Datta GD, Lauzon M, Salvy SJ, Hussain SK, Ghandehari S, Merchant A (2022). Cancer Screening Practices Among Healthcare Workers During the COVID-19 Pandemic. Front Public Health.

[56] Hange N, Agoli AM, Lourdes Pormento MK, Sharma A, Somagutta MR (2022). Impact of COVID-19 response on public health literacy and communication. Health Promot Perspect.

[57] Siegel RL, Miller KD, Fuchs HE, Jemal A (2022). Cancer statistics, 2022. CA Cancer J Clin.

[58] Burzic A, O'Dowd EL, Baldwin DR (2022). The Future of Lung Cancer Screening:Current Challenges and Research Priorities. Cancer Manag Res.

[59] Delva F, Laurent F, Paris C, Belacel M, Brochard P, Bylicki O, Chouaïd C (2019). LUCSO-1-French pilot study of LUng Cancer Screening with low-dose computed tomography in a smokers population exposed to Occupational lung carcinogens:study protocol. BMJ Open.

[60] Dement JM, Ringen K, Hines S, Cranford K, Quinn P (2020). Lung cancer mortality among construction workers:implications for early detection. Occup Environ Med.

[61] Veronesi G, Baldwin DR, Henschke CI, Ghislandi S, Iavicoli S, Oudkerk M (2020). Recommendations for Implementing Lung Cancer Screening with Low-Dose Computed Tomography in Europe. Cancers (Basel).

[62] Hiatt RA, Beyeler N (2020). Cancer and climate change. Lancet Oncol.

[63] Nogueira LM, Yabroff KR, Bernstein A (2020). Climate change and cancer. CA Cancer J Clin.

[64] Andrews O, Le Quéré C, Kjellstrom T, Lemke B, Haines A (2018). Implications for workability and survivability in populations exposed to extreme heat under climate change:a modelling study. Lancet Planet Health.

[65] He C, Zhang Y, Schneider A, Chen R, Zhang Y, Ma W, Kinney PL, Kan H (2022). The inequality labor loss risk from future urban warming and adaptation strategies. Nat Commun.

[66] Gronlund CJ (2014). Racial and socioeconomic disparities in heat-related health effects and their mechanisms:a review. Curr Epidemiol Rep.

[67] Hinchliffe A, Kogevinas M, Pérez-Gómez B, Ardanaz E, Amiano P, Marcos-Delgado A (2021). Occupational heat exposure and breast cancer risk in the MCC-Spain study. Cancer Epidemiol Biomarkers Prev.

[68] Vineis P, Huybrechts I, Millett C, Weiderpass E (2021). Climate change and cancer:converging policies. Mol Oncol.

[69] Persson L, Carney Almroth BM, Collins CD, Cornell S, de Witt CA, Diamond ML (2022). Outside the Safe Operating Space of the Planetary Boundary for Novel Entities. Environ Sci Technol.

[70] Vrijheid M, Casas M, Bergström A, Carmichael Am, Cordier, Effesbo M (2012). European birth cohorts for environmental health research. Environ Health Perspect.

[71] Turner MC, Mehlum IS (2018). Coordination and Harmonisation of European Occupational Cohorts is Needed. Occup Environ Med.

[72] COST (2017). Strategic Plan COST 060/17.

[73] Kogevinas M, Schlünssen V, Mehlum IS, Turner MC (2020). The OMEGA-NET international inventory of occupational cohorts. Ann Work Expo Health.

[74] Peters S, Vienneau D, Sampri A, Turner MC, Castaño G, Bugge M (2022). Occupational exposure assessment tools in Europe:a comprehensive inventory overview. Ann Work Expo Health.

[75] Atkas E, Bergbom B, Godderis L, Kreshpaj B, Marinov M, Mates D (2022). Migrant workers occupational health research:an OMEGA-NET working group position paper. Int Arch Occup Environ Health.

[76] Guseva Canu I, Marca SC, Dell'Oro F, Balazs A, Bergamaschi E, Besse C (2021). Harmonized definition of occupational burnout:A systematic review, semantic analysis, and Delphi consensus in 29 countries. Scand J Work Environ Health.

[77] Pramesh CS, Badwe RA, Bhoo-Pathy N, Booth CM, Chinnaswamy G, Dare AJ (2022). Priorities for cancer research in low- and middle-income countries:a global perspective. Nat Med.

[78] GBD (2021). 2019 Respiratory Tract Cancers Collaborators Global, regional, and national burden of respiratory tract cancers and associated risk factors from 1990 2019:a systematic analysis for the Global Burden of Disease Study 2019. Lancet Respir Med.

[79] Sepanlou SG, Etemadi A, Kamangar F, Sepehr A, Pourshams A, Poustchi H (2013). The gastro-esophageal malignancies in Northern Iran research project:impact on the health research and health care systems in Iran. Arch Iran Med.

[80] Sheikh M, Shakeri R, Poustchi H, Pourshams A, Etemadi A, Islami F (2020). Opium use and subsequent incidence of cancer:results from the Golestan Cohort Study. Lancet Glob Health.

[81] Veljkovikj I, Ilbawi AM, Roitberg F, Luciani S, Barango P, Corbex M (2022). Evolution of the joint International Atomic Energy Agency (IAEA), International Agency for Research on Cancer (IARC), and WHO cancer control assessments (imPACT Reviews). Lancet Oncol.

